# Plant Nitrogen Assimilation: A Climate Change Perspective

**DOI:** 10.3390/plants14071025

**Published:** 2025-03-25

**Authors:** Mirwais M. Qaderi, Cameryn C. Evans, Madeleine D. Spicer

**Affiliations:** Department of Biology, Mount Saint Vincent University, 166 Bedford Highway, Halifax, NS B3M 2J6, Canada; cameryn.evans@dal.ca (C.C.E.); madeleine.spicer3@msvu.ca (M.D.S.)

**Keywords:** climate change, C_3_ and C_4_ plants, elevated carbon dioxide, higher temperature, water stress, nitrogen assimilation, protein, abiotic stress, interactive abiotic stressors

## Abstract

Of all the essential macronutrients necessary for plant growth and development, nitrogen is required in the greatest amounts. Nitrogen is a key component of important biomolecules like proteins and has high nutritive importance for humans and other animals. Climate change factors, such as increasing levels of carbon dioxide, increasing temperatures, and increasing watering regime, directly or indirectly influence plant nitrogen uptake and assimilation dynamics. The impacts of these stressors can directly threaten our primary source of nitrogen as obtained from the soil by plants. In this review, we discuss how climate change factors can influence nitrogen uptake and assimilation in cultivated plants. We examine the effects of these factors alone and in combination with species of both C_3_ and C_4_ plants. Elevated carbon dioxide, e[CO_2_], causes the dilution of nitrogen in tissues of non-leguminous C_3_ and C_4_ plants but can increase nitrogen in legumes. The impact of high-temperature (HT) stress varies depending on whether a species is leguminous or not. Water stress (WS) tends to result in a decrease in nitrogen assimilation. Under some, though not all, conditions, e[CO_2_] can have a buffering effect against the detrimental impacts of other climate change stressors, having an ameliorating effect on the adverse impacts of HT or WS. Together, HT and WS are seen to cause significant reductions in biomass production and nitrogen uptake in non-leguminous C_3_ and C_4_ crops. With a steadily rising population and rapidly changing climate, consideration must be given to the morphological and physiological effects that climate change will have on future crop health and nutritional quality of N.

## 1. Nitrogen Assimilation in a Changing Climate

Nitrogen (N) is a component of many critical biochemical molecules, including proteins, nucleic acids, and chlorophyll, which are essential to the structure, function, and growth of plants. Correspondingly, the demand for N in plants is the highest of all minerals [1]. Plants also require varying amounts of energy for nutrient uptake and assimilation, with N assimilation involving some of the most energy expensive biochemical reactions of all [1]. Plants mainly acquire N in the form of inorganic nitrate (NO_3_^−^), ammonium (NH_4_^+^), and ammonium derived from the degradation of urea (CO(NH_2_)_2_) [2]. These inorganic forms of N are then assimilated following a sequence of reactions: first, the absorbed NO_3_^−^ is reduced to nitrite (NO_2_^−^) by the enzyme nitrate reductase (NR); second, NO_2_^−^ is reduced to NH_4_^+^ by the enzyme nitrite reductase (NiR); third, NH_4_^+^ is incorporated into amino acids through the action of the enzyme glutamine synthetase (GS) and subsequently glutamine synthase (GOGAT). GS converts NH_4_^+^ into glutamine, while GOGAT converts glutamine into glutamate, which then serves as a precursor for the synthesis of other amino acids. It should be mentioned that the assimilation process requires this sequence of reductions and incorporations and is not limited only to the enzymes mentioned [1]. These amino acids are the main form of N transport in plants and are then used in various metabolic processes. They are transported via the xylem to vegetative tissues for use in photosynthesis and protein synthesis, while other amino acids are transported via phloem to storage tissues, like roots and developing seeds [3]. More recent evidence has shown that plants are also capable of directly taking up organic forms of N, such as amino acids or proteins [4,5]. Atmospheric nitrogen (N_2_) is converted to plant-available NO_3_^−^ and NH_4_^+^ and deposited in the soil by means of both the biological processes of N-fixing symbiotic bacteria and industrial techniques, such as the Haber–Bosch process, for the production of NH_3_-based fertilizers [6]. Microorganisms in the soil help supply NO_3_^−^ and NH_4_^+^ to the soil by the biodegradation of organic matter and the fixation of N_2_ through a series of biochemical reactions involved in N fixation, ammonification, and nitrification [7]. Through nitrification, soil microbes oxidize NH_4_^+^ to nitrite and then further oxidize NO_2_^−^ to NO_3_^−^, which can be absorbed and assimilated into organic N-containing compounds by plants [8]. The absorption of NO_3_^−^ and NH_4_^+^ is facilitated by proteins of the nitrate transporter (NRT) family and the ammonium transporter (AMT) family, respectively [9].

N also functions as an important signaling molecule, and its presence or absence can activate different N-mediated signaling pathways. N-starvation responses (NSRs) are induced when limited amounts of N are present, affecting gene expression, which activates increased uptake and assimilation for N by NRTs and a drive to use organic N sources. When sufficient N is again sensed by plants, the primary nitrate responses (PNRs) are activated, controlling nitrate-responsive gene expression via a Ca_2_^+^ signal cascade [10]. NO_3_^−^ also functions as a signal molecule in plants; it is controlled by many genes and has impacts on N use efficiency (NUE), thereby affecting outcomes like plant growth, grain development, and crop yield. *OsNRT2.3b*, for example, increases N uptake, improving grain yield and NUE, when overexpressed in rice (*Oryza sativa* L.) [11]. Environmental factors have been shown to both positively and negatively impact these processes.

Since the industrial revolution, the atmospheric CO_2_ concentration ([CO_2_]) has been increasing rapidly, with a current concentration of 424.61 ppm in 2024 [12] and a projected increase of between 500 and 700 ppm by the year 2100 [13]. As CO_2_ accumulates, the mean global temperature increases as well. In fact, the global temperature has increased by 0.85–1.3 °C from 1880 to 2012 and is expected to further increase by 1.5 °C (with more than 50% likelihood) to representative concentration pathways (RCPs) that exceed 4 °C (RCP 8.5) by the end of this century, depending on emission scenarios and the prediction model used [13]. Soil moisture and drought have also been projected to worsen with global temperature increase, with the most significant effect on the more arid regions of the globe [13]. As climate change worsens, it is important to evaluate how it will influence plant productivity and quality. The global population is expected to reach 9.8 billion by 2050 [14]; therefore, a substantial increase in crop supply will be needed to meet the increasing food demand [14]. Accordingly, the individual effects of climate change factors, namely elevated carbon dioxide (e[CO_2_]), higher temperature (HT), and drought stress (water stress, WS, hereafter), on plant N uptake and assimilation have been studied extensively. However, the relative effects of these factors, both individually and in combination, on N assimilation in C_3_ species (which include all legumes and many other plants/crops) and C_4_ species are underrepresented in the literature. Humans and other animals require large amounts of these plants in their diets to fuel the need for N; therefore, it is important to consider how environmental factors will influence future N accumulation and utilization in cereal crops and legumes, especially amid a rapidly changing climate.

This review aims to: (i) integrate and compare the known effects of climate change factors (elevated carbon dioxide concentration, e[CO_2_]; higher temperature, HT; water stress, WS) on C_3_ and C_4_ plant N uptake and assimilation and (ii) make predictions on how these climate change factors may undermine or improve future crop health and productivity.

## 2. Effects of Elevated Carbon Dioxide

**Figure 1:**
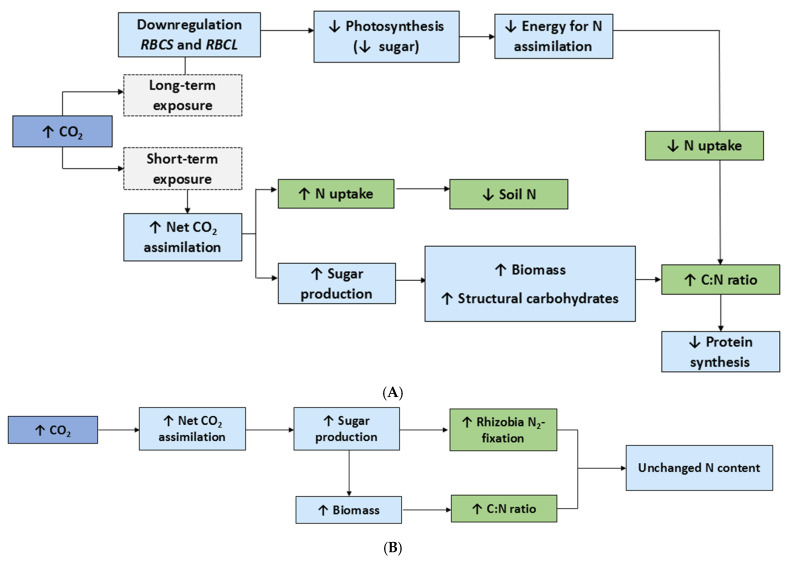
The effects of long-term and short-term exposure to elevated CO_2_ in (**A**) non-leguminous C_3_ and C_4_ plants and (**B**) leguminous C_3_ plants. Upward arrows, increase; downward arrows, decrease; RBCS, small subunit of RuBisCO; RBCL, large subunit of RuBisCO. Effects on plants are based on the literature cited in Table 1.

Of all the climate change factors considered in this review, the bulk of the research appears to focus on e[CO_2_]. Respectively, evidence that e[CO_2_] increases plant biomass production is extensive [15]. This is true across leguminous and non-leguminous C_3_ and C_4_ species (see Table 1), with the most significant improvement of growth seen in C_3_ plants [16]. At ambient CO_2_ (a[CO_2_]) levels, C_3_ plants are at risk of photorespiration, as oxygen (O_2_) competes with CO_2_ in binding to the active site of RuBisCO [17]. Photorespiration is pronounced under stress conditions (e.g., higher temperature and water stress) because these environmental stressors lead to the closure of stomata, limiting the amount of CO_2_ available for photosynthesis [18]. Thus, in C_3_ plants grown under e[CO_2_] conditions, the internal leaf CO_2_:O_2_ ratio is higher, favoring photosynthesis and reducing photorespiration [19]. It has also been shown that e[CO_2_] results in an overall decline in plant N concentration over time and, in turn, leads to a decline in protein content (see Figure 1) [20].

A well-known case of this e[CO_2_]-induced protein decline was shown in wheat (*Triticum aestivum* L.) [21,22,23,24], where e[CO_2_] increased grain yield by 11.4% and N accumulation by 12.9% at the 50% anthesis and by 9.2% at the ripening stage across the three years of the study [23]. In these studies, however, e[CO_2_] decreased protein concentration [21,22,23,24]. The physiological cause for the decrease in N content remains largely unknown and is under some debate (see [25] and references therein). A common speculation is that the rate at which N is supplied to the soil is not efficient enough to keep pace with the rate of N uptake by the plant under e[CO_2_] conditions. Likewise, under e[CO_2_], C_3_ plants generally grow and accumulate non-structural carbohydrates faster than N is acquired, consequently increasing the C:N ratio [26]. This phenomenon is known as the dilution effect [25,27], and it can have negative effects on plants, as less N will be available for amino acid and protein synthesis in the growing plant. This idea is supported by numerous studies (see Table 1). As in most non-leguminous C_3_ plants, C_3_ legumes, including soybean (*Glycine max* L.) [28], common bean (*Phaseolus vulgaris* L.) [29], and chickpea (*Cicer arietinum* L.) [30], all exhibit an increased biomass production when grown under e[CO_2_]. For instance, e[CO_2_] increased chickpea yield by 21.1% and 21.9% in the first and second years of study, respectively; however, there was no change in seed N content [30].

It is well documented that plants obtain carbon via aboveground leaf photosynthesis and nitrogen nutrients through underground root absorption. The ability of sensing, transporting, assimilating, and utilizing carbon and N between source and sink organs is important for plant growth and survival [31]. As shown, plants possess a regulatory machinery that can coordinate N assimilation with carbon metabolism, nutrient availability, and other environmental factors [31,32], maintaining homeostasis for carbon- and N-related processes in above- and below-ground tissues [32].

**Table 1:** Effects of individual environmental factors (e[CO_2_], HT, WS) on growth/biomass production, N uptake, and total N and protein content of non-leguminous C_3_ and C_4_ plants and leguminous C_3_ plants.

Environmental Factor	Common Name	Scientific Name	Growth/Yield/Biomass Production	Total N Content	N Uptake	Protein	Experimental Condition	References
**Non-leguminous C_3_**								
e[CO_2_]	Wheat	*Triticum aestivum* L.	↑	↓	↑	NM	FACE	[23]
	Wheat	*Triticum aestivum* L.	↑	↓	↑	NM	FACE	[22]
	Wheat	*Triticum aestivum* L. cv. Yipti	NM	NM	↑	↓	FACE	[24]
	Wheat	*Triticum aestivum* L. genotypes, BTS and GE	LN ↑ shoot, ↓ root HN ↑ shoot, ↓ root	LN - HN ↓	LN ↑ HN ↓	NM	Growth chamber	[33]
	Wheat	*Triticum aestivum* L. “Ayahikari”	↑	↓	↑	NM	Growth chamber	[34]
	Rice	*Oryza sativa* L. “Nipponbare”	↑	↓	↑	NM	Growth chamber	[34]
	Potato	*Solanum tuberosum* L. “Irish Cobbler”)	↑	↓	↑	NM	Growth chamber	[34]
	Rice	*Oryza sativa* L. japonica “Kitaake” Wild Type and OsCV Silenced	*OsCV* Silence ↑ > WT ↑	As amino acid *OsCV* Silence − WT ↓	*OsCV* Silence ↑ WT ↓	*OsCV* Silence ↑ WT↓	Growth chamber	[35]
HT	Canola	*Brassica napus* L., cv. 6056	↓	NM	NM	NM	Growth chamber	[36]
	Tomato	*Solanum lycopersicum* L.	↓	↓	↓	↓ N uptake proteins	Growth chamber	[37]
	Wheat	*Triticum aestivum* L.	↓	NM	NM	NM	Growth chamber	[38]
WS	Barley	*Hordeum vulgare* L.	↓	↓	↓	↓	Greenhouse	[39]
	Sweet potato	*Ipomoea batatas* (L.) Lam. cv. Xushu 32 and Ningzishu 1	↓	↑ NO_3_^−^ shoots ↓ NO_3_^−^ roots NH_4_^+^ -	↓	↑ NR leaves ↓ NR roots	Greenhouse	[40]
**Non-leguminous C_4_**								
e[CO_2_]	Corn	*Zea mays* L.	↑	↓	↑	NM	Open-top chamber	[41]
	Guinea grass	*Panicum maximum* Jacq. “Natsukaze”	↑	↓	↑	NM	Growth chamber	[34]
	Amaranth	*Amaranthus* spp. L. (Tusrushin seeds, Co., Ltd., Japan)	↑	-	↑	NM	Growth chamber	[34]
HT	Corn	*Zea mays* L.	↓	↑	NM	↑	Open-top chamber	[42]
	Waxy corn	*Zea mays* L. sinensis Kulesh cv. Suyunuo 5	↓	NM	↓	↓	Greenhouse	[43]
WS	Corn	*Zea mays* L.	↓	↓	↓	↓	Greenhouse	[39]
	Corn	*Zea mays* L.	↓	↓	↓	NM	Greenhouse	[44]
	Corn	*Zea mays* L.	NM	↑	↑ NH_4_^+^	NM	Greenhouse	[9]
**Leguminous C_3_**								
e[CO_2_]	Chickpea	*Cicer arietinum* L.	↑	-	↑	NM	Field	[30]
	Common bean	*Phaseolus vulgaris* L.	↑	↑	NM	NM	Growth room	[29]
	Soybean	*Glycine max* L.	↑	-	↑	NM	FACE	[28]
HT	Common bean	*Phaseolus vulgaris* L.	↓	↓	NM	↓	Growth chamber	[45]
	Mung bean	*Vigna radiata* L.	↑	↑ NBI	NM	NM	Growth chamber	[46]
WS	Alfalfa	*Medicago sativa* L.	↓	NM	NM	↓	Field	[47]
	Soybean	*Glycine max* (L.) Merr. cv. Shennong17, Shennong8, Shennong12	↓	↓	↓	↓	Field	[48]

a[CO_2_], ambient carbon dioxide; aT, ambient temperature; e[CO_2_], elevated carbon dioxide; FACE, free-air CO_2_ enrichment; HN, high nitrogen supplementation; HT, higher temperature; LN, low nitrogen supplementation; NBI, nitrogen balance index; NM, not measured; NR, nitrogen reductase; WS, water stress; WW, well-watered; >, greater than; ↑, increased; ↓, decreased; -, no significant effect.

A study revealed that plants that showed no increase in growth or biomass due to e[CO_2_] exhibited lowered concentrations of N in their tissues, while plants that had increased biomass in response to e[CO_2_] were found to have no decrease in the quantities of N [49]. Another study found that e[CO_2_] had varying effects on wheat plants depending on the N supply conditions and genotype, causing changes in gene expression responsible for N assimilation in leaves and roots [33]. For e[CO_2_], the enzymes for NO_3_^−^ and NH_4_^+^ utilization are downregulated; for e[CO_2_] × low N conditions, N storage is increased; for e[CO_2_] × high N conditions, N storage is decreased. This indicates that e[CO_2_] influences the enzyme and gene expression regarding N uptake and storage, with root N storage less affected than shoot N storage (Table 1) [33]. A recent study on canola (*Brassica napus* L.) also showed that e[CO_2_] increased net [CO_2_] assimilation but did not lead to an increase in growth or biomass. The authors suggest that plant metabolites might have been used to ameliorate the effects of stressors (e.g., temperature) rather than used for growth [36].

A study comparing the impacts of a[CO_2_] and e[CO_2_] on a C_3_ plant, wheat, with a C_4_ plant, corn (*Zea mays* L.), showed that e[CO_2_] concentrations positively impacted the C_3_ plant’s ability to utilize NH_4_^+^ as a N source [50]. For e[CO_2_], wheat had increased CO_2_ assimilation, allowing the formation of more carbon skeletons, which allowed NH_4_^+^ to be assimilated more efficiently, reducing NH_4_^+^-induced stress. This effect was not significant in C_4_ corn [50]. Since NH_4_^+^ can be used by plants more efficiently than NO_3_^−^, due to not requiring further biochemical reduction by enzymatic activity, e[CO_2_] may make fertilization by urea-based fertilizers more efficient and feasible in C_3_ crops [50]. Another study on corn showed that biomass and N uptake increased under e[CO_2_], while total N content decreased [41].

The hypothesis that the N_2_-fixing capacity of leguminous species may serve to combat negative impacts of environmental stressors on N assimilation is well supported in the literature [51,52]. For example, while the N content of wheat typically decreases when grown at e[CO_2_], that of the C_3_ legume chickpea remains relatively unchanged [30]. Similar results were obtained in another study of the effects of e[CO_2_] on nutrient uptake in soybean, where the biomass and N uptake increased significantly, while the overall N content was not affected (Table 1) [28]. Many earlier studies have shown that e[CO_2_] can increase total N content in legumes because of a stronger symbiosis between the plant and *Rhizobia*, whereby higher CO_2_ allows for increased production of carbohydrates that can be exchanged for more N [53]. This was also seen in a more recent study on common bean (Table 1) [29]. In contrast, regarding a[CO_2_], C_4_ leaves are saturated with CO_2_ due to their specialized leaf anatomy and metabolism; thus, the effects of increasing CO_2_ on C_4_ biomass often tend to not be as robust [54]. However, C_4_ plants grown in e[CO_2_] conditions have still been shown to exhibit an increased biomass production and show some degree of N dilution (Table 1) [42]. Stimulation of growth under e[CO_2_] typically does not last very long, as plants may eventually acclimate to the new conditions, or growth will be limited by nutrient availability, such as that of N [15]. In fact, RuBisCO has also been shown to be diminished in the leaves of plants grown under longer periods of e[CO_2_] exposure [27], which ultimately suppresses photosynthesis. In durum wheat (*Triticum durum* Desf.), genes encoding both the small subunit (*RBCS*) and the large subunit (*RBCL*) of RuBisCO were downregulated in e[CO_2_] conditions, which is thought to be directly related to constraints to N uptake, as N is one of the major components of RuBisCO [55]. The downregulation of gene expression associated with nutrient transport from the root system has also been observed under e[CO_2_] conditions in C_3_ plants [49], which exhibit photorespiration.

*Photorespiration* is an essential component of photosynthesis [1] that occurs primarily in C_3_ plants and is closely linked with NO_3_^−^ assimilation [16,56]. As mentioned above, e[CO_2_] inhibits photorespiration in C_3_ plants, as the CO_2_:O_2_ ratio is high within the leaves; thus, RuBisCO favors its carboxylase function over oxygenase, consequently reducing NO_3_^−^ assimilation [57,58]. The inhibition of NO_3_^−^ assimilation in e[CO_2_] conditions has been shown in Arabidopsis (*Arabidopsis thaliana* (L.) Heynh.), wheat, and barley (*Hordeum vulgare* L.) [59]. Since NO_3_^−^ requires more energy for assimilation than NH_4_^+^, plants grown strictly under NO_3_^−^ nutrition may be more negatively affected by climate change factors that limit energy production. Bloom et al. [60] suggested that NO_3_^−^ assimilation is inhibited in the roots of wheat and Arabidopsis in e[CO_2_] conditions, while Andrews et al. [29] showed that the assimilation of N was similar whether plants received NO_3_^−^ or NH_4_^+^. More research should be conducted here to find consistent evidence. Bloom et al. [60] also showed that root NO_3_^−^ assimilation increased and shoot NO_3_^−^ assimilation decreased in the C_3_ plants wheat and Arabidopsis in e[CO_2_] conditions because e[CO_2_] inhibited malate production in chloroplast [60].

A study using an Arabidopsis wild-type (Col-0) and a mutant defective in peroxisomal hydroxy-pyruvate reductase (*hpr1-1*) that was hampered in photorespiratory turnover showed that the reason for the reduction in N content in e[CO_2_] conditions was due to acclimation causing a reduction in photorespiration in e[CO_2_] conditions, leading to a decline in NO_3_^−^ assimilation. During photorespiration under a[CO_2_], N is sunk into amino acids, eventually leading to the provision of carbon skeletons for further N assimilation. In e[CO_2_] conditions, this process is decreased and N assimilation declines [61]. Conversely, due to the CO_2_ concentrating mechanism of C_4_ species, photorespiration occurs at much lower rates [62]. However, the first reaction in the C_4_ pathway, in which phosphoenolpyruvate carboxylase (PEPcase) binds CO_2_, produces large amounts of malate and NADH in the cytoplasm of mesophyll cells, supporting NO_3_^−^ assimilation [16].

Overall, an increase in CO_2_ is expected to benefit the biomass production of C_3_ plants, as the carboxylation of RuBisCO will be favored, enhancing sugar production and growth. However, with an increasing biomass comes a greater demand for N to produce amino acids, proteins, and other biomolecules; thus, in a nutrient-poor environment, an increased biomass may not be sustainable and can have harmful consequences, including a decrease in N content (see Table 1). These effects may be curbed in legumes by increasing N_2_ fixation by bacteria housed in their root nodules if sufficient phosphorus is present to sustain the production of adenosine triphosphate (ATP) for nitrogenase activity [52,63].

Igarashi et al. [49] showed that, when biomass increased due to e[CO_2_] levels being lower than anticipated, it was not due to the e[CO_2_] inhibition of NO_3_^−^ assimilation [34]. They studied this in C_3_ plants using wheat, rice, and potatoes and C_4_ plants using Guinea grass (*Panicum maximum* Jacq.) and amaranth (*Amaranthus* spp. L.). Rice was the only species to show a slight decrease in NO_3_^−^ storage in e[CO_2_] conditions. The findings showed that e[CO_2_] had a dramatic impact on water-use efficiency (WUE), increasing it compared to a[CO_2_], particularly in the C_3_ species. This was not due to a reduction in transpiration from e[CO_2_] but was due to more efficient use of water for CO_2_ capture. The hypothesis that e[CO_2_] reduced N in the plant due to overall increases in biomass was confirmed and further tested in wheat and Guinea grass grown under e[CO_2_] by administering varying levels of NO_3_^−^ or urea-sourced N during growth. The growth rate of C_4_ Guinea grass was not affected, but the C_3_ wheat growth rate was increased by e[CO_2_], even with limited NO_3_^−^. The maximum relative growth rate (RGR) in wheat in e[CO_2_] conditions required 1.3-times the N required for a[CO_2_], yielding 2.2-times the biomass. The authors concluded that e[CO_2_] causes the pace of biomass growth to outstrip the plant ability to uptake and store N in N-deficient environments but does not inhibit the plant’s actual N uptake and storage abilities if sufficient N is available (Table 1) [34].

Transcriptomic research efforts have documented various genes, many of them part of the NRT encoding gene families, whose expression is associated with variations in N uptake under e[CO_2_] in *Arabidopsis* and wheat [49]. The identification of these genes suggests that genetic modification and manipulation may be one possible method to render crops better suited to adapt to the challenges of a changing climate, along with careful supplemental N administration [49]. For example, a study knocking out the gene *OsCV* in rice, which controls chloroplast vesiculation, was shown to increase N assimilation and protein under e[CO_2_] conditions (Table 1) [35].

Again, as suggested by the IPCC [13], an increase in the atmospheric concentration of CO_2_ will come with an increase in global temperature; thus, the effects of increased temperature should also be of heavy consideration when evaluating how climate change affects plant N assimilation.

## 3. Effects of High Temperature

Major changes in atmospheric temperature have been predicted to lead to periods of prolonged higher-than-baseline temperatures and drought in some regions of the world [1]. Under high temperatures, plants are at risk of water loss due to transpiration, which causes closure of the stomata to minimize dehydration through abscisic acid (ABA) signaling [64]. When stomata are closed in C_3_ plants, CO_2_ cannot enter the leaves, and RuBisCO cannot readily catalyze CO_2_ fixation for photosynthesis, leading to a higher risk of photorespiration. Furthermore, the solubility of CO_2_ in water also decreases more relative to that of O_2_ under HT, further favoring photorespiration and negatively affecting plant growth as a result [16]. Conversely, the leaf anatomy seen in C_4_ plants may provide an advantage under HT; since CO_2_ is more concentrated within the leaves, they can keep their stomata closed for longer periods, preventing desiccation under temperature stress, while still favoring photosynthesis over photorespiration [65]. As temperature affects metabolic rate and enzyme kinetics, HT might also actually increase the rate of photosynthesis in plants [66]. Other studies have shown a decrease in photosynthetic rate, with HT reducing the efficiency of photosynthetic enzymes, leading to a decline in net CO_2_ assimilation, plant growth, and biomass (Table 1) [36]. However, as seen in many plants grown at e[CO_2_] levels, the rate of photosynthesis may be too fast for the plant, thereby shortening the window of time available for the efficient accumulation and utilization of resources during development. A recent study on wheat showed that HT at critical stages of development significantly decreases photosynthetic rates and grain yields per plant, due to thylakoid membrane damage. In this study, HT during anthesis and grain filling decreased the rate of photosynthesis by 17% and 25%, respectively, and grain yield per plant by 29% and 44%, respectively (Table 1) [38].

**Figure 2:**
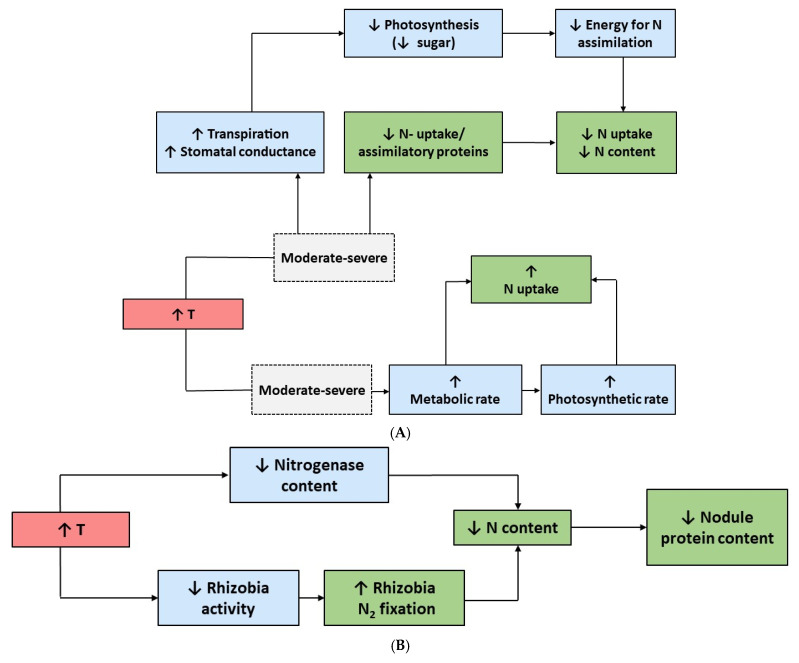
The effects of elevated temperatures in (**A**) non-leguminous C_3_ and C_4_ species and in (**B**) leguminous C_3_ plants. Upward arrows, increase; downward arrows, decrease; T, temperature. Effects on plants are based on the literature cited in Table 1.

Heat stress tends to have negative effects on nutrient assimilation in most plants (see Figure 2 and Table 1) [37]. Many plants can acclimate to small changes in temperature, but more extreme temperature changes can downregulate and/or damage important enzymes and proteins involved in assimilation, as seen in *Arabidopsis* [67]. Studies have shown that genes coding for N transporters and enzymes involved in N assimilation are downregulated in response to increasing temperature [68]. It is well documented that nitrate reductase activity is drastically reduced under HT in plants [69].

A study on Arabidopsis showed that HT has a drastic effect on the expression of many genes, including genes that code for stress responses. Heat treatment induced the expression of 1107 genes and reduced the expression of 697 genes [70]. The authors concluded that the genes whose expression was most affected by HT were those that were most widespread in expression. The downregulation of genes for proteins used in photosynthesis, for example, may allow plants to put more energy into stress adaptation responses. Genes that encode for the degradation and recycling of chloroplast proteins can also be up-regulated by HT. This redirection of gene expression away from the basic functions of energy production could explain some of the reduced N assimilation, and thus negative plant growth and protein outcomes, seen under HT [70]. A study on waxy corn (*Zea mays* L. sinensis Kulesh) investigated the effects of HT on grain yield. The authors found that HT decreased overall biomass and grain yield, as well as reduced the activity of nitrate reductase (NR) and glutamine synthetase (GS) enzymes. This indicates a decrease in N metabolism that negatively impacted the grain yield (Table 1) [43].

In cytosol, NR catalyzes the reduction of accumulated NO_3_^−^ to NO_2_^−^, which is subsequently reduced to NH_4_^+^ by nitrite reductase (NiR) in plastids, and then, it is used to synthesize N-containing metabolites like amino acids [71]. Thus, N assimilation is generally negatively affected when plants are grown in HT. In support of this, in a study of the effects of HT stress on Indian mustard (*Brassica juncea* L.), genes encoding NR (*BjNR1* and *BjNR2*) and other important proteins, such as GS, NRTs, and AMTs, were mainly downregulated [43]. Similarly, in the roots of tomato (*Solanum lycopersicum* L.) grown under moderate and severe HT stress, NRT1 (low-affinity NO_3_^−^ transporter), NRT2 (high-affinity NO_3_^−^ transporter), and AMT1 (high-affinity NH_4_^+^ transporter) levels all decreased relative to the controls [37]. Additionally, levels of NR, glutamate synthase (GOGAT), glutamine dehydrogenase (GDH), and GS all experienced a similar pattern, and the overall biomass production and tissue N concentration decreased compared to the controls [37]. Contrasting with these results, a separate study showed that, when plants were grown under HT, N-uptake rates were slightly accelerated in tomato seedlings, leading to an overall increase in total N [72]. From these results, it is likely that N-uptake may decline only if warming exceeds the optimal temperature of N-uptake proteins, as seen in studies mentioned above. In leguminous species, it has been shown that both NO_3_^−^ uptake and symbiotic N_2_ fixation decrease when plants are grown under high-temperature stress (Table 1) [45]. In response to HT stress, the common bean exhibited significant decreases not only in nitrogenase and root nodule protein content, but also in NR, GOGAT, and GS [45]. Additionally, because the symbiosis between legumes and *Rhizobia* is susceptible to temperature, temperatures outside of the bacterial optimal range can have deleterious effects on N_2_ fixation and the overall N and protein content of the legume [73].

Generally, an increase in temperature above the optimal temperature range of plants has harmful effects on plant N uptake and assimilation. In leguminous C_3_ and non-leguminous C_3_ and C_4_ plants, HT tends to cause the downregulation of important enzymes, as well as transport and assimilatory proteins involved in N assimilation [37], which is likely at least partly responsible for the reduced productivity and nutritional quality of plants grown under HT.

## 4. Effects of Water Stress

**Figure 3:**
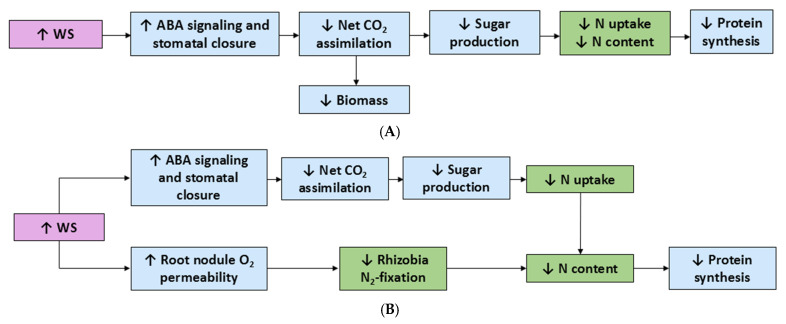
The effects of water-deficient water stress in (**A**) non-leguminous C_3_ and C_4_ plants and (**B**) leguminous C_3_ plants. Upward arrows, increase; downward arrows, decrease; WS, water stress. Effects on plants are based on the literature cited in Table 1.

Of all the resources that plants require for growth, development, and function, water is both the most abundant in the biosphere and frequently the most limiting. The uptake of nutrients from the soil is dependent on its water content; for plants to obtain nutrients, there must be an adequate amount of water in which the nutrients can dissolve, be taken up by, and transported within the plant. In the soil, nutrients can move to the root surface by both diffusion and mass flow [1]. An early response by plants to water stress is stomatal closure via ABA signaling from the root system, which functions to minimize water loss to the atmosphere [18,74]. Closing of the stomata under increased ABA signaling in water stress inhibits the diffusion of CO_2_ into the leaf for photosynthesis and has been correlated with reduced growth, disturbance of nutrient uptake, and oxidative stress (Table 1) [46,75]. With inadequate water availability and energy production through decreased photosynthesis, plants can be adversely affected (see Figure 3). Accordingly, NO_3_^−^ and NH_4_^+^ uptake and assimilation have been shown to decrease under WS in many plants, perhaps due to a limited amount of available energy [75].

NO_3_^−^ also functions as an important signaling molecule, along with water, in crosstalk that could cause changes in what genes are expressed by plants in response to WS conditions [76]. For example, Han et al. [77] found that, in mutant rice strains, when the gene *OsNR1.2* is deactivated, the mutant rice endures WS better than wild-type rice cultivars [77]. *OsNR1.2* is a gene involved in N assimilation that is controlled by a C_2_H_2_ zinc-finger transcription factor called DST (drought and salt tolerance). Under WS, DST expression is reduced, leading to the downregulation of the *OsNR1.2* gene, inhibiting N uptake and assimilation and increasing WS tolerance. Since knocking out *OsNR1.2* increases WS tolerance even further, this shows that a reduction in N assimilation is a key part of the physiological strategy for coping with WS in rice [77].

A decrease in N uptake, total N content, and protein has been demonstrated in C_3_ crop barley and in C_4_ crop corn (Table 1) [39]. This decrease in N and protein is also seen in many legumes whereby WS increases the permeability of root nodules to oxygen, thereby disrupting the anaerobic conditions required for N_2_ fixation and, consequently, reducing N content, protein production, and overall grain yield [74,78]. This was seen in C_3_ legume alfalfa (*Medicago sativa* L.), where both the yield and crude protein concentration decreased when grown in severe WS conditions (Table 1) [47].

Since NO_3_^−^ uptake and assimilation consumes much more energy than that of NH_4_^+^, the effects of WS on NH_4_^+^ assimilation may not be as severe [79]. In support of this, recent studies have consistently shown that AMTs in Chinese cottonwood (*Populus simonii* Carrière) [80], corn [9], and barley [39] are often upregulated in response to drought, while NRTs are downregulated (Table 1). For instance, in Chinese cottonwood grown under WS, NRTs were downregulated, while AMTs were upregulated, leading to a significant decrease in NO_3_^−^ but only a small decrease in NH_4_^+^ [80]. In another study where Chinese crab-apple trees (*Malus prunifolia* (Willd.) Borkh) were subjected to WS, the transcript abundance of most NRTs was suppressed, while that of AMTs was increased [75]. Another study on sweet potato (*Ipomoea batatas* (L.) Lam. cv. Xushu 32 and Ningzishu 1) also showed the deleterious effects of WS on biomass, N metabolism, and N assimilation [40]. Plant growth and biomass were negatively affected; NO_3_^−^ levels increased in leaves but decreased in roots, while NH_4_^+^ levels remained stable, altering the overall plant ratios of NO_3_^−^ to NH_4_^+^ concentrations. The levels of NR also increased in leaves but decreased in roots. Overall, the plants suffered more of a decrease in shoot biomass than root biomass under WS, and a downregulation of genes like *NRT1* was observed (Table 1) [40]. Studies in soybean have also shown that biomass and N metabolism are negatively affected by long exposures to WS. There was an observed downregulation of genes responsible for N metabolism, like *GmNR*, *GmNiR*, *GmGs*, and *GmGOGAT*, and a decrease in enzymatic activities for N metabolism. Overall, this led to plants having a decrease in biomass and N and protein concentrations, with an increase in NO_3_^−^ concentrations in their leaves (Table 1) [48].

In contrast, AMTs and NRTs have both shown to be upregulated in barley and corn when grown in WS conditions. In this study, WS decreased total plant N by 45% and 44% in barley and corn, respectively [39]. A study on corn showed that WS enhanced almost all genes involved in N uptake in the roots, including the genes for AMTs, NRTs, NR, and GS, and increased N-uptake and amino acid concentrations [9]. The upregulation of these genes may be because N plays a major role in the alleviation of water stress in plants [81,82]. Solute accumulation is a known mechanism for drought tolerance in plants. When grown under WS, metabolites, including amino acids and proteins, often accumulate in plant tissues [83]. The extent of nitrogenous solute accumulation by means of N assimilation typically depends upon the amount of available N in the environment. Under WS, plants tend to allocate N to water-soluble nitrogenous compounds in the tissues, where their osmoregulatory properties will help counteract desiccation [82]. Photosynthetic N-use efficiency (PNUE) and the allocation of N in various plant tissues are changed to help plants compensate under WS conditions, depending on the availability of N. In a study on rice under WS, PNUE and the storage of N in leaf tissue varied from high to low N availability [84]. Under high N and WS, rice plants decreased N allocation into photosynthetic leaf tissue components and increased N allocation in non-photosynthetic leaf tissue. Under low N conditions and WS, plants decreased N in the cell walls and the photosynthetic light harvesting system. This balancing act, as well as an increase in solutes like protein and free amino acids, allowed the rice plants to maintain PNUE under WS conditions [84].

The accumulation of N-containing compounds may serve as an explanation for the upregulation of AMTs. One such amino acid that often accumulates is proline, which has osmotic adjusting and protective properties [85]. The accumulation of proline has been linked to the regulation of enzymes responsible for the synthesis of proline from glutamine, namely D1-pyrroline-5-carboxylate synthetase (P5CS) and D1-pyrroline-5-carboxylate reductase (P5CR) [86,87]. In many plant species, including *Arabidopsis* [86] and barley [88], water deprivation causes the upregulation of genes encoding these two enzymes.

## 5. Interactive Effects of Elevated CO_2_, High Temperature, and Water Stress

We have discussed how individual climate change factors influence plant N uptake and assimilation. However, in real environmental conditions, multiple abiotic factors interact simultaneously, affecting plants collectively. Therefore, the combined effects of e[CO_2_], HT, and WS should be considered. Here, we summarize the main findings from earlier studies of the interactive effects of climate change factors on the uptake and assimilation of N in crops and make predictions about the effects of these factors where necessary.

### 5.1. Combined Effects of Elevated CO_2_ and High Temperature

As increasing CO_2_ and temperature are correlated, several studies have been conducted to evaluate their combined effects on crops. As noted, e[CO_2_] and HT can individually have deleterious effects on C_3_ crops, and accordingly, many studies have shown that the effects of e[CO_2_] and HT are additive in their negative effects. In one study, while both e[CO_2_] and severe HT independently had minimal influence on biomass production in tomato seedlings, e[CO_2_] combined with HT severely restricted their growth (Table 2) [72]. In the same experiment, while seedlings grown under either e[CO_2_] or HT only underwent slight changes in N-uptake proteins and total N concentration, those grown under combined e[CO_2_] and severe HT exhibited a significant decrease in the activity of AMT1, concentrations of NRT1, NR, GS, and GOGAT, and overall N uptake [72]. Building on this previous research, Jayawardena et al. [89] showed that e[CO_2_] × chronic HT negatively impacted the growth of tomatoes (*Solanum lycopersicum* L. cv. Big Boy), affecting the uptake of NO_3_^−^ and NH_4_^+^, with NO_3_^−^ uptake most severely affected [89]. The severe effect on NO_3_^−^ uptake, rather than on NH_4_^+^, may be primarily responsible for the overall decrease in N assimilation in the plant. This reduction also led to a significant decline in the proteins required for N uptake in the roots. Net N movement, the overall movement of N within the plant between roots and shoots, was shown to decrease under e[CO_2_] × HT by the N and NO_3_^−^ ratios between the plant shoots and roots. Under e[CO_2_] and HT conditions, the overall transport of N from roots to shoots decreased, as indicated by changes in the ratios of total N and NO_3_^−>^ between these plant parts. N assimilation was shown to be affected in two main ways: (1) by alterations in the balance between mineral N (such as NO_3_^−>^) and organic N (such as N in amino acids and proteins) and (2) by changes in the distribution of N between the total N pool in the plant and the N incorporated into proteins. Moreover, the authors concluded that the reduced N rates of uptake and absorption were not due to insufficient energy or resources in the tomato seedlings’ roots (Table 2) [89]. Similarly, a decrease in N assimilation under e[CO_2_] and HT, denoted by a decrease in amino acid and soluble protein concentrations, has been shown in wheat and is thought to be related to a decline in energy available to sustain NR activity through the reduction in RuBisCO carboxylation activity (Table 2) [58]. In rice, e[CO_2_] and HT decreased grain yield by 18–29% and protein content by 4–6% compared to e[CO_2_] alone (Table 2) [90]. In another study on a tropical rice cultivar, e[CO_2_] and HT increased aboveground dry mass by 84.5% but decreased the grain yield advantage by 3% from the increased yield of 22.6% by e[CO_2_] alone over a three-year period. In grains, e[CO_2_] also increased NUE (Table 2) [91].

Although C_4_ species are not affected by e[CO_2_] and HT individually, the negative effects of e[CO_2_] and HT on N uptake and assimilation are not limited to C_3_ species. C_4_ crops tend not to be as responsive as C_3_ crops to e[CO_2_]; therefore, we might predict that plant growth under e[CO_2_] and HT—where HT is within the optimal temperature range of N-assimilation proteins—may prevent the dilution effect and increase the overall N content of C_4_ crops. More research is needed on this topic in order to draw conclusions.

Some studies have suggested a buffering effect between e[CO_2_] and HT, whereby e[CO_2_] partially alleviates the negative effects of HT on biomass (Table 2) [102]. A recent study on the effects of N supplementation and HT, e[CO_2_], and HT × e[CO_2_] showed that canola plants suffered more negative effects from HT than from e[CO_2_], and that when grown with supplemental urea (CO(NH_2_)_2_), some of the negative effects of these stressors, particularly HT, were alleviated (Table 2) [36]. For example, elevated CO_2_ alleviated the negative impact of heat stress on wheat photosynthesis and biomass but not on its grain yield [103]. In another study, Chavan et al. showed that, in wheat, e[CO_2_] can negatively impact photosynthetic capacity with a long duration of exposure; it still increases biomass and grain yield but causes lower N and protein concentrations in the grain (Table 2) [93]. Under a[CO_2_] × HT conditions, no significant impact was seen on grain yield and biomass; however, e[CO_2_] × HT negated the increasing effects of e[CO_2_] on yield and biomass in WW conditions, despite enhancing photosynthesis [93].

A recent study on canola also examined the combined effects of e[CO_2_] and HT with N supplementation. Individually, plants grown with e[CO_2_] or HT displayed poorer growth than plants grown under control conditions, with overall reduced biomass due to fewer leaves and thinner stems. Plants grown under e[CO_2_] × HT conditions had the most negatively affected growth. The authors reported that the lowest biomass occurred under e[CO_2_]× HT, contradicting the idea that e[CO_2_] mitigates the effects of HT. Supplemental NO_3_^−^ benefitted the plants more than supplemental NH_4_^+^ regardless of e[CO_2_] or temperature, with supplemental NO_3_^−^ increasing photosynthesis [104].

In a study, the spring wheat plants grown at e[CO_2_] levels had increased growth irrespective of elevated or optimal temperature conditions, but plants grown under both e[CO_2_] and HT conditions had the lowest concentrations of N in their tissues (Table 2) [92]. It is believed that N dilution is responsible for the low tissue levels of N, as the increased growth caused by e[CO_2_] outpaces N tissue storage capabilities under HT × e[CO_2_], although the N uptake rate per unit root appears unaffected [92]. These findings predict that, as wheat crops are grown under the increasing stressors of HT × e[CO_2_], the protein concentrations of the grain are lowered, impacting its value and quality as a food crop [92].

Legumes also appear to display this buffering effect in their biomass production [105]. This is likely due to the increased allocation of C to the root nodules, which would potentially increase N_2_ fixation and, in turn, protein content. This is apparent in one study where soybeans grown under a[CO_2_] and HT exhibited decreased protein content and biomass, while growing them under the combination of e[CO_2_] and HT resulted in protein content being no different from the controls (Table 2) [99]. However, studies on the effects of e[CO_2_] combined with HT on legume N uptake and assimilation are sparse, and more research is required to determine how these factors influence N uptake and assimilation, as well as N_2_ fixation, within the root nodules.

### 5.2. Combined Effects of Elevated CO_2_ and Water Stress

The effects of CO_2_ combined with WS on plant N uptake and assimilation are extremely limited in the literature; thus, predictions must be made. As noted previously, N uptake and assimilation in C_3_ plants generally increases under e[CO_2_] because of more available energy, while the opposite is true under WS. Given that e[CO_2_] and WS generally have opposite effects on photosynthesis—increase and decrease, respectively—these factors in combination may have antagonistic effects on each other in terms of the plant response (see Table 2). Correspondingly, it has been shown in many studies that e[CO_2_] can help offset the negative effects of WS on C_3_ and C_4_ plants (see [106] and references therein). In fact, e[CO_2_] helps alleviate WS by improving photosynthetic rates and leaf water status [107]. Interestingly, it has also been shown that C_4_ crops only tend to benefit from growth under e[CO_2_] when WS causes a decrease in *g***_s_**, increase in WUE, and increase in leaf internal [CO_2_] [54]. This supports an early study on C_4_ crop sugarcane (*Saccharum officinarum* L. cv. CP72-2086), where e[CO_2_] reduced the effects of WS by decreasing *g*_s_ and improving water-use efficiency [108]. Moreover, an early study on C_3_ crop barley showed that growth under simultaneous exposure to e[CO_2_] and WS resulted in increased NR activity and NH_4_^+^ assimilation relative to those grown under WS alone [109].

In another study on barley, biomass was decreased by WS, while it was increased by e[CO_2_]; e[CO_2_] × WS saw a decline in biomass that was not as great as the decline caused by WS alone, indicating a protective effect on biomass accumulation during drought by the e[CO_2_] (Table 2) [95]. Total plant N concentration was lowered by both WS and e[CO_2_] and found to be lowest in the e[CO_2_] × WS treatment. N uptake and root proteins were also lowest in the e[CO_2_] × WS treatment; e[CO_2_] alone increased both N uptake and root proteins, while WS decreased them, again showing overall a protective effect by the e[CO_2_] during WS conditions, although the effect was not as drastic as on biomass [95]. The effects of CO_2_ and WS on different genotypes of durum wheat have shown that e[CO_2_] combined with moderate WS tended to consistently upregulate the transcripts for GS1, suggesting an increase in N assimilation [55]. In contrast, in one study on sweet pepper (*Capsicum annuum* L.), e[CO_2_] in combination with WS resulted in an overall decrease in amino acid concentration, pointing to a decrease in N assimilation (Table 2) [94]. As for legumes, in a study on soybean, e[CO_2_] appeared to revert the expression of WS-induced genes mainly related to processes such as transport and nutrient deficiency, e.g., iron (Table 2) [100]. Knowing this, the effects of these factors on processes involved in N transport should also be investigated in future studies.

One research team genetically altered a rice cultivar, “IR64”, to manifest fewer stomata by overexpressing Epidermal Patterning Factor *OsEPF1*; these lines of *OsEPF1* rice cultivars were better able to cope under e[CO_2_] × WS than control cultivars of IR64, using between 38 and 42% less water than control plants [110]. The final 1000 grain mass between control and genetically altered plants indicated that expressing fewer stomata under drought conditions whilst flowering is protective for future grain yield [110]. Currently, there is a deficit of information on the combined effects of e[CO_2_] and WS on non-leguminous C_4_ plants. Although the individual effects of these stressors have been studied, more research is called for to clarify the impacts that will occur on some of the common crops of rice and corn as carbon dioxide concentrations rise and drought worsens [111].

### 5.3. Combined Effects of High Temperature and Water Stress

It has been estimated that approximately half of the yield losses in crops are a result of damage caused by HT and WS [112]. The rates of combined incidents of drought and heatwave are only expected to increase as climate change worsens [13]. Depending on species, the stress responses to HT and WS individually tend to have opposing physiological mechanisms, and the combination of the two stresses can be devastating. The response of opening stomata to dissipate heat during HT is directly opposed by the response of stomatal closure to conserve water loss during WS [113]. These factors appear to have similar effects across most C_3_ and C_4_ species, in that, both individually and in combination, they can cause water loss, disturbance of nutrient uptake and enzyme activity, and an overall reduction in photosynthetic activity and biomass production (see Table 1 and Table 2). Not surprisingly, it appears that these effects only worsen when the two factors act on plants simultaneously.

A study on soybean showed that HT × WS caused a decline in photosynthetic activity, stomatal conductance, free amino acid content, and electron transport activity (Table 2) [101]. Notably, HT and WS conditions resulted in a more marked decrease in overall N content, which led to lower concentrations of soluble proteins and free amino acids, which would otherwise act in maintaining osmotic balance (Table 2) [96]. A study in which corn hybrids were subjected to HT and WS showed that, while corn exhibited an increase in proline concentration, the concentration of soluble protein, as well as nitrogen uptake, decreased drastically (Table 2) [44]. A study by Ru et al. [97] on winter wheat (*Triticum aestivum* L. cultivar Xiaoyan 22′) also showed that the combined stressors of HT × WS caused a reduction in NR activity that became more severe the longer the duration of exposure to the treatment, limiting N uptake and storage (Table 2) [97]. HT × WS also decreased both shoot and root dry biomass; this effect has been shown to lead to increased losses during climate stress in wheat [97]. Further studies by Ru et al., examining the mitigating role of N supply on the effects of HT × WS, showed that high N application negatively affected wheat plants, increasing the negative effects of the combined stressors, while moderate N application can improve growth outcomes in HT or WS treatments alone [114].

As mentioned previously, HT and WS can be especially detrimental to plants, as they can negatively influence plant stress responses to environmental factors. For example, while HT and WS individually typically result in ABA signaling in the plant to close the stomata and conserve water, several studies have shown that HT can work to oppose the ABA-inducing effects of WS, meaning more water loss [102,115,116].

At times, HT can benefit plants in some ways by speeding up their metabolism. However, research suggests that warming is only advantageous to plants when there is enough water available to support growth and alleviate the water loss that occurs through temperature-mediated transpiration (Table 2) [98]. Thus, it is likely that HT in a water-limited environment will have deleterious effects on N assimilation and plant productivity. This is supported in an early study in which NR and GS activities significantly decreased in Chinese ryegrass (*Leymus chinensis* (Trin.) Tzvelev) when grown under a combination of moderate-to-severe water stress and above-ambient temperatures [96]. Similarly, in a study on C_4_ crop Guinea grass, while HT under irrigated conditions (WW) increased biomass production and N concentration, warming combined with WS caused a decrease in total N and biomass (Table 2) [98]. WS × HT have also been shown to decrease leaf area, plant height, dry mass, and seed production in C_3_ Tartary buckwheat (*Fagopyrum tataricum* L.), with the severity of the effects dependent on the variety. The combined stressors had a worse effect than each individual stressor [117]. Thus, HT and WS appear to be additive in their negative effects on crops rather than opposing each other (Table 2) [44]. One study on alpine grass (*Poa hothamensis *var. *hothamensis* N.G. Walsh) indicated that WS can induce better tolerance of subsequent heat stress; although, total dry biomass was significantly negatively affected in the treated plants [118]. The effects of combined WS and HT appear to be partly species-specific [118].

HT and WS have also been shown to significantly decrease total N content and root nodule activity in legumes through diminished nitrogenase concentration (Table 3) [119]. We recognize that there is only a very small amount of evidence regarding these effects on N uptake and assimilation in legumes; nevertheless, we make the prediction that legume N assimilation will be negatively influenced by severe HT and WS, as both factors alone tend to upset key players in the assimilatory process (i.e., temperatures outside of *Rhizobia* optimal range, disruption of nodule anaerobic conditions, etc.) (Table 2) [101]. Overall, more research is needed to examine the mechanisms by which HT and WS, in combination, influence growth as well as N uptake dynamics and assimilation in crops, with a focus on leguminous species.

### 5.4. Combined Effects of Elevated CO_2_, High Temperature, and Water Stress

To the best of our knowledge, there are very limited studies that explore the combined effects of all three climate change factors on plant N uptake and assimilation. In this review, only one study, using Arabidopsis, measured N assimilation under the three stress conditions, considering amino acid and protein content. However, based on the available studies and evidence cited above, some speculations can be made for the combined effects of these factors on plant N uptake, accordingly (see Table 3). In some species, e[CO_2_] has been shown to mitigate the negative effects on C_3_ crops caused by HT and WS, perhaps by altering stomatal conductance and/or photosynthetic rates. For instance, an early study on wheat showed that the decrease in biomass production caused by drought was partially mitigated when grown under e[CO_2_] and elevated temperature [120]. Comparably, a recent study showed that e[CO_2_] can work to alleviate damaging oxidative stress caused by HT and WS in Arabidopsis [67]. This is consistent with results from another study where e[CO_2_] also appeared to decrease oxidative stress caused by HT and WS in both grasses and legumes, such as perennial ryegrass (*Lolium perenne* L.) and black medic (*Medicago lupulina* L.), thereby supporting a greater biomass production relative to those in the HT × WS conditions [121].

A study on various genotypes of tomato examined the effects of e[CO_2_] and a[CO_2_] and the effects of e[CO_2_] × HT × WS and a[CO_2_] × HT × WS treatments. The study showed that HT × WS had a significant deleterious effect regardless of [CO_2_] concentration (Table 3) [122]. Plants exposed to e[CO_2_] × HT × WS had a higher net photosynthetic rate than plants grown under the a[CO_2_] × HT × WS treatment. Testing of e[CO_2_] and a[CO_2_] showed that e[CO_2_] increased *g*_s_ over a[CO_2_] when not combined with the other stressors. In combination, however, e[CO_2_] had some protective effect against HT × WS by lowering *g*_s_ and increasing WUE, compared to plants receiving the a[CO_2_] × HT × WS treatment. This protective effect appeared to vary based on genotype, indicating that some cultivars may do better than others in e[CO_2_] × HT × WS environments [122].

**Table 3:** Effects of three-way interactions of environmental factors (e[CO_2_] × WS × HT) on growth/biomass production, N uptake, and total N and protein content of non-leguminous C_3_ and C_4_ plants and leguminous C_3_ plants.

Environmental Factors	Common Name	Scientific Name	Growth/Yield/Biomass Production	Total N Content	N Uptake	Amino Acids	Protein	Experimental Condition	References
**Non-leguminous C_3_**									
e[CO_2_] × HT × WS	Canola	*Brassica napus* L. cv. 45H72	e[CO_2_] × HT ×WS > a[CO_2_] × HT × WS	NM	NM	NM	NM	Growth chamber	[102]
	Arabidopsis	*Arabidopsis thaliana* L.	NM	NM	NM	HT × WS > e[CO_2_] × WS × HT	↓	Climate chamber	[123]
	Tomato	*Solanum lycopersicum* L. “OuBei” and *Solanum pimpinellifolium* L. ‘LA2093’	e[CO_2_] × HT ×WS > a[CO_2_] × HT × WS	NM	NM	NM	NM	Climate chamber	[122]
e[CO_2_] × HT × WS	Sorghum	*Sorghum bicolor* L.	e[CO_2_] × HT × WS > a[CO_2_] × HT × WS	NM	NM	NM	NM	Greenhouse	[124]
**Leguminous C_3_**									
e[CO_2_] × HT × WS	Alfalfa	*Medicago sativa* L.	e[CO_2_] × WS × HT > a[CO_2_] × WS × HT	-	NM	NM	NM	Growth chambers	[119]

a[CO_2_], ambient carbon dioxide; e[CO_2_], elevated carbon dioxide; HT, higher temperature; NM, not measured; WS, water stress; >, greater than; ↓, decreased; -, no significant effect.

A study conducted on the C_4_ crop sorghum (*Sorghum bicolor* L.), evaluating the combined effects of e[CO_2_] × HT × WS, confirmed that e[CO_2_] caused an increase in biomass without the stressors of HT and WS, while a treatment of e[CO_2_] × HT × WS showed that e[CO_2_] had no effects on photosynthesis but did prevent a loss of biomass due to HT and WS (Table 3) [124]. Sorghum exposed to HT × WS and a[CO_2_] suffered a loss of biomass, due in part to greater *g*_s_ from the HT at a[CO_2_], which may cause additional WS to the plant; e[CO_2_] appeared to reduce *g*_s_, offering protection against this additional WS and thus protecting biomass through better WUE, despite a reduction in photosynthetic activity under the combined stressors [124]. In contrast, it was also shown that, in Arabidopsis, amino acids accumulated more strongly when exposed to HT, WS, and a[CO_2_] relative to those grown under similar conditions of HT and WS, but at e[CO_2_] levels (Table 3) [123]. In the same study, the key genes related to nitrate uptake, translocation, and assimilation were down-regulated by the stress factors [123].

**Figure 4:**
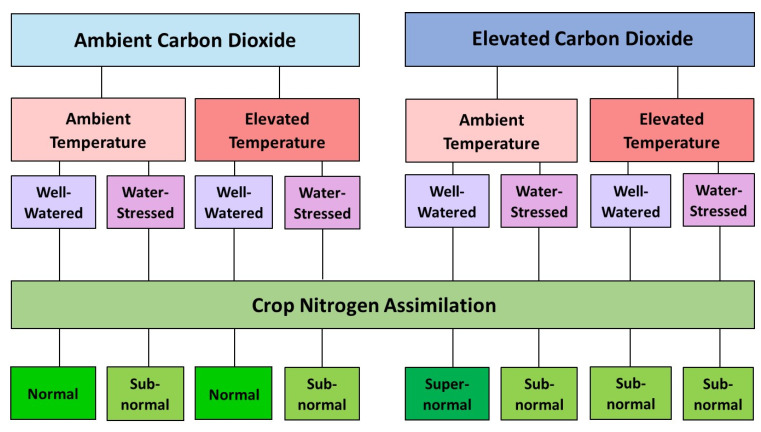
A flowchart of the interactive effects of [CO_2_], temperature, and watering status on N assimilation in C_3_ and C_4_ plants. Effects on plants are based on the literature cited in Table 2 and Table 3.

Overall, the interactive effects of e[CO_2_], HT, and WS on crops appear to be highly dependent on the species at hand and the severity of each individual factor (see Figure 4). Based on earlier studies, we speculate that a combination of e[CO_2_], HT, and WS can affect C_3_ and C_4_ crop N uptake and assimilation. Further research is necessary on this topic.

## 6. Concluding Remarks and Future Perspectives

Both individually and in combination, the stressful effects of climate change factors on plants depend to a large degree on the type of plant and severity of the stress. Firstly, while the more primitive C_3_ plants tend to benefit under short-term e[CO_2_] exposure, C_4_ plants generally exhibit little to no advantage due to their existing specialized CO_2_ concentrating mechanism. In non-leguminous plants, long-term e[CO_2_] exposure tends to decrease the overall concentration of N because of a dilution effect, where the C:N ratio increases. Legumes appear to bypass this N dilution in part because of their N-fixing abilities, although there is a lack of research on the effects of individual or combined abiotic stressors in C_4_ leguminous species. Secondly, mild-to-moderate HT stress can speed up N assimilation in some cases, but much of the literature suggests that moderate-to-severe cases of HT cause the downregulation of important assimilatory proteins and decreases photosynthetic rates in C_3_ and C_4_ plants, thereby decreasing enzymes and energy required for sustained N assimilation. Above-optimal temperatures can also disrupt the symbiotic relationship between leguminous plants and *Rhizobia* housed in their root nodules. Interestingly, CO_2_ has been shown to alleviate some of the consequences of HT stress on biomass production; however, studies must be expanded to explore how N assimilation is influenced under the same conditions. Thirdly, WS indirectly and directly negatively affects N assimilation in numerous ways. Most notably, it causes the closure of stomata and a decrease in photosynthesis, which subsequently decreases the energy available for N assimilation. Furthermore, WS makes legume root nodules more permeable to O_2_, upsetting the anaerobic conditions required for N_2_ fixation by *Rhizobia*. While the negative effects of WS are generally worsened when combined with HT stress, e[CO_2_] can act to counteract them in some cases. At present, research is very limited when it comes to the simultaneous effects of all three climate change factors on plant N assimilation.

Taking all this information into account, the responses of crops will surely differ from one region to another. As crop productivity is more closely linked to local climate changes rather than global climate changes, crops in one region will perform better relative to another due to variations in climate across the globe [116]. Nevertheless, with the global climate changing at such an alarming rate, action must be taken immediately. As the atmospheric carbon dioxide concentration increases, the rate of photosynthesis increases, causing an increase in the C:N ratio in plants. Accordingly, humans and other animals will need to consume more plants to sustain their needs for N. Moreover, the increasing atmospheric temperature and declining water availability pose major threats to plant N content. These facts, paired with the rapidly rising population, point to the urgency of research that should be conducted and initiatives that must be taken to give possible solutions for battling the impacts of these climate change factors on important plant processes like N assimilation. Solutions might include the implementation of more strict emission guidelines worldwide, judicious science-based fertilization practices, and the development of genetic varieties of plants with resistance to harsh environmental conditions so that they can adapt more readily to the changing environment. To accomplish the latter, future studies should place more focus on how climate change factors alter gene expression involved in N assimilation. It has been suggested that the modification of N metabolism, using biotechnological methods, may lead to increased biomass accumulation and yield in crop plants. Additionally, it is possible to evaluate the improvement of NUE by using transgenic plants. Therefore, the application of genetic engineering and genome editing can help mitigate the effects of climate change on N assimilation by improving NUE in crops [125].

## Figures and Tables

**Table 2:** Effects of the two-way interactions of environmental factors (e[CO_2_] × HT, e[CO_2_] × WS, HT × WS) on growth/biomass production, N uptake, and total N and protein content of non-leguminous C_3_ and C_4_ plants and leguminous C_3_ plants.

Environmental Factor	Common Name	Scientific Name	Growth/Yield/Biomass Production	Total N Content	N Uptake	Protein	Experimental Condition	References
**Non-leguminous C_3_**								
e[CO_2_] × HT	Sula wheat	*Triticum durum* Desf. cv. Sula	e[CO_2_] × HT > HT	HT > e[CO_2_] × HT	NM	HT > e[CO_2_] × HT	Greenhouse	[58]
	Tomato	*Solanum lycopersicum* L.	HT > e[CO_2_] × HT	HT > e[CO_2_] HT	HT > e[CO_2_] × HT	HT > e[CO_2_] × HT	Greenhouse	[72]
	Tomato	*S. lycopersicum* L. cv. Big Boy	HT > e[CO_2_] × HT	HT > e[CO_2_] × HT	HT > e[CO_2_] × HT	HT > e[CO_2_] × HT	Greenhouse	[89]
	Rice	*Oryza sativa* L. var. Nerica-L-44 and Pusa 1121	e[CO_2_] > e[CO_2_] × HT	NM	NM	e[CO_2_] > e[CO_2_] × HT	Open-top chamber	[90]
	Rice	*Oryza sativa* L., cv. Naveen	e[CO_2_] × HT > e[CO_2_] -	a[CO_2_] > e[CO_2_] × HT	e[CO_2_] × HT > a[CO_2_]	NM	Open-top chamber	[91]
	Wheat	*Triticum aestivum* L.	e[CO_2_] × HT = e[CO_2_]	e[CO_2_] > e[CO_2_] × HT	e[CO_2_] × HT -	e[CO_2_] > e[CO_2_] × HT	Greenhouse	[92]
	Wheat	*Triticum aestivum* L. cv Scout and Yipti	e[CO_2_] > e[CO_2_] × HT a[CO_2_] × HT -	e[CO_2_] < a[CO_2_] Yipti	NM	e[CO_2_] < a[CO_2_]	Greenhouse	[93]
	Canola	*Brassica napus* L., cv. 6056	e[CO_2_] × HT × LN > e[CO_2_] × HT × ZN	NM	NM	NM	Growth chamber	[36]
e[CO_2_] × WS	Sweet pepper	*Capsicum annuum* L.	e[CO_2_] × WS > WS	WS > e[CO_2_] × WS	NM	NM	Greenhouse	[94]
	Barley	*Hordeum vulgare* L.	e[CO_2_] > e[CO_2_] × WS > WS	e[CO_2_] > WS > e[CO_2_] × WS	e[CO_2_] > e[CO_2_] × WS > WS	e[CO_2_] > e[CO_2_] × WS > WS	Greenhouse	[95]
HT × WS	Chinese rye grass	*Leymus chinensis* Trin.	HT, WS > HT × WS	HT, WS > HT × WS	NM	HT, WS > HT × WS	Greenhouse	[96]
	Wheat	*Triticum aestivum* L. cultivar Xiaoyan 22′	HT, WS > HT × WS	HT, WS > HT × WS	HT, WS > HT × WS	HT, WS > HT × WS NR levels	Growth chamber	[97]
**Non-leguminous C_4_**								
HT × WS	Corn	*Zea mays* L.	HT, WS > HT × WS	HT, WS > HT × WS	HT, WS > HT × WS	WS > HT, HT × WS	Greenhouse	[44]
	Guinea grass	*Panicum maximum* Jacq.	HT × WS > HT, WS	HT, WS > HT × WS	NM	NM	T-FACE	[98]
**Leguminous C_3_**								
e[CO_2_] × HT	Soybean	*Glycine max* L.	e[CO_2_] > e[CO_2_] × HT	a[CO_2_] > e[CO_2_] × HT	NM	-	Open-top chamber	[99]
e[CO_2_] × WS	Soybean	*Glycine max* L.	e[CO_2_] × WS > WS	NM	NM	NM	Open-top chamber	[100]
HT × WS	Soybean	*Glycine max* L.	HT, WS > HT × WS	NM	NM	NM	Greenhouse	[101]

a[CO_2_], ambient carbon dioxide; aT, ambient temperature; e[CO_2_], elevated carbon dioxide; FACE, free-air CO_2_ enrichment; HN, high nitrogen supplementation; HT, higher temperature; LN, low nitrogen supplementation; NBI, nitrogen balance index; NM, not measured; NR, nitrogen reductase; T-FACE, temperature free-air controlled enhancement; WS, water stress; WW, well-watered; ZN, zero nitrogen supplement; >, greater than; -, no significant effect.

## Data Availability

Not applicable.
